# Facet-Related Non-uniform Photoluminescence in Passivated GaAs Nanowires

**DOI:** 10.3389/fchem.2020.607481

**Published:** 2020-12-07

**Authors:** Nian Jiang, Hannah J. Joyce, Patrick Parkinson, Jennifer Wong-Leung, Hark Hoe Tan, Chennupati Jagadish

**Affiliations:** ^1^Electrical Engineering Division, Engineering Department, University of Cambridge, Cambridge, United Kingdom; ^2^Department of Physics and Astronomy, The Photon Science Institute, University of Manchester, Manchester, United Kingdom; ^3^Department of Electronic Materials Engineering, Research School of Physics, The Australian National University, Canberra, ACT, Australia; ^4^Australian Research Council (ARC) Centre of Excellence for Transformative Meta-Optical Systems, Research School of Physics, The Australian National University, Canberra, ACT, Australia

**Keywords:** nanowire, photoluminescence (PL), nanowire sidewall facets, surface recombination, uniformity, GaAs-AlGaAs

## Abstract

The semiconductor nanowire architecture provides opportunities for non-planar electronics and optoelectronics arising from its unique geometry. This structure gives rise to a large surface area-to-volume ratio and therefore understanding the effect of nanowire surfaces on nanowire optoelectronic properties is necessary for engineering related devices. We present a systematic study of the non-uniform optical properties of Au-catalyzed GaAs/AlGaAs core–shell nanowires introduced by changes in the sidewall faceting. Significant variation in intra-wire photoluminescence (PL) intensity and PL lifetime (τ_*PL*_) was observed along the nanowire axis, which was strongly correlated with the variation of sidewall facets from {112} to {110} from base to tip. Faster recombination occurred in the vicinity of {112}-oriented GaAs/AlGaAs interfaces. An alternative nanowire heterostructure, the radial quantum well tube consisting of a GaAs layer sandwiched between two AlGaAs barrier layers, is proposed and demonstrates superior uniformity of PL emission along the entire length of nanowires. The results emphasize the significance of nanowire facets and provide important insights for nanowire device design.

## Introduction

Semiconductor nanowires have been widely considered as one of the prime candidates for future devices, benefiting from their geometry that promises to overcome many of the challenges caused by lattice mismatch, and adding new functionality to devices (Lieber and Wang, 2007; Wong-Leung et al., 2019). Over last few decades, tremendous progress has been made in the materials science of semiconductor nanowires, and in their application in electronic and optoelectronic devices such as photovoltaics (Czaban et al., 2009; Tang et al., 2011; Parkinson et al., 2013; Li et al., 2018), lasers (Saxena et al., 2013, 2016; Eaton et al., 2016; Koblmüller et al., 2017), photodetectorsm (Lapierre et al., 2017; Gibson et al., 2019), THz detectors (Peng et al., 2015, 2020), LEDs (Koester et al., 2015), transistors (Tomioka et al., 2012), etc. Despite successful demonstrations, the performance of nanowire solar cells are still far below that predicted by simulation and are yet to match their thin film counterparts (Goktas et al., 2018). Nanowire THz detectors show particular advantages for future devices, such as on-chip applications, when size or spatial resolution is critical (Nagel et al., 2002; Cunningham et al., 2010; Peng et al., 2020). Nevertheless, further device optimization is still needed to improve the signal-to-noise ratio (Peng et al., 2020).

Since the performances of nanowire devices are determined by the fundamental material properties in the active region, achieving their full potential requires nanowires of uniform and as high optoelectronic quality as a planar material (Parkinson et al., 2013). Studies on planar structures have shown that the electrical and chemical properties are highly crystal-orientation dependent (Wang, 1986; Meney, 1992; Chand, 1993). This surface-related effect is even more profound for nanowires due to their high surface-to-volume ratio. The quasi-one-dimensional geometry and complicated sidewall facets behavior (Jiang et al., 2014) makes nanowires very vulnerable to non-uniform properties along their length. In fact, non-uniform behaviors are commonly observed in nanowires (Spirkoska et al., 2009; Thunich et al., 2009; Demichel et al., 2010; Chang et al., 2012; Parkinson et al., 2013; Bolinsson et al., 2014). Various reasons have been reported to cause this non-uniformity, including diameter changes (Tomioka et al., 2012), unintentional radial growth (Thunich et al., 2009), crystal phase structure changes (Spirkoska et al., 2009; Bolinsson et al., 2014), doping variations (Alanis et al., 2019) and incomplete surface passivation (Chang et al., 2012). However, the performances of nanowire devices are still below predictions (Goktas et al., 2018) even with all the suggested causes eliminated.

Here we report a new mechanism based on nanowire sidewall orientations that leads to non-uniform optical properties of Au-catalyzed GaAs/AlGaAs core–shell nanowires. We also suggest new structures to achieve uniform properties along the nanowire.

## Experiment

GaAs/AlGaAs core–shell nanowires were grown on GaAs (111)B substrates by metal organic chemical vapor deposition (MOCVD). Trimethylgallium (TMGa), trimethylaluminum (TMAl), and arsine (AsH_3_) were used as the sources for Ga, Al and As, respectively. Au nanoparticle colloids with diameters of 100 and 250 nm were chosen as catalysts, and their diameters determined the diameter of the grown GaAs nanowire core. Hereafter, nanowires are identified by the approximate diameter of their GaAs core, as either “100 nm cores” or “250 nm cores.” The GaAs cores were grown using a two-temperature procedure to minimize the unintentional radial growth and produce twin-free zinc-blende nanowires (Joyce et al., 2007). The temperature was ramped up to 750°C for AlGaAs shell growth with a total group III flow rate of 1.5 × 10^−5^ mol/min, [TMAl]/[TMAl + TMGa] = 0.5 and V/III ratio [AsH_3_]/[TMAl + TMGa] of 100, resulting in Al concentration between 0.4 and 0.45 (Shi et al., 2015). The AlGaAs shell was grown for 3 min to obtain a thick enough barrier to passivate the surface of GaAs core nanowires (Jiang et al., 2013). For the quantum well tube nanowires, TMAl was switched off for the growth of a 2.5 nm GaAs quantum well tube growth, followed by another 3 min AlGaAs layer growth. A thin GaAs capping layer was finally deposited around the nanowires to protect the AlGaAs shell from oxidation. This capping layer has negligible impact on the photoluminescence measurement, owing to fast non-radiative recombination at its surface that occurs within the first 20 ps after photoexcitation (Joyce et al., 2014). Therefore, we disregard the GaAs cap layer in the following analysis.

The GaAs core nanowires show a background doping <1 × 10^15^ cm^−3^ (Joyce et al., 2013). For the core–shell nanowires, the growth resulted in an AlGaAs shell uniformly covering the GaAs core nanowire (Jiang et al., 2013). The AlGaAs shell thicknesses were studied by scanning transmission electron microscope (JOEL 2100F) on cross-sectional samples. The results show that AlGaAs shell thicknesses increase with GaAs core diameters, with the thinnest shell thickness at 16 nm for nanowires with 50 nm GaAs core nanowires (Supplementary Figure 1). Under these circumstances, the impact of AlGaAs shell thickness on the surface passivation is negligible as it has been shown that the shell growth time rather than shell thickness (>16 nm) determines the interface quality (Jiang et al., 2013).

The GaAs/AlGaAs core–shell nanowires were transferred onto Si substrate for photoluminescence (PL) measurements at room temperature in air. PL permits spatially-resolved measurements of the optoelectronic properties of single nanowire in a contact-free fashion. Being contact-free, it avoids the artifacts caused by device fabrication and measurement, and circumvents the well-known challenges associated with making ohmic contacts to undoped GaAs nanowires (Wirths et al., 2012). A 522 nm solid state pulsed laser was used, providing 0.2–14 μJ/(pulse^*^cm^2^) fluence within a 0.5 μm focal spot after a 100× objective lens. This arrangement provides a spatial resolution of around 0.5 μm for spatially-resolved PL. A low excitation power was used to avoid band filling and band renormalization effects. The emitted PL signal was detected by a monochromator and a cooled CCD detector. PL was collected in two configurations, (1) with a pinhole placed in front of the monochromator to limit the PL collection area to a length of 1 μm centered at the excitation spot, and (2) without the pinhole which permits PL to be collected from the entire nanowire. Unless otherwise specified, PL signals were collected from the whole nanowire. PL lifetimes (τ_PL_) were measured at the emission peak by a single photon avalanche photodiode and a time correlated single photon counting (TCSPC) system. The time-resolved PL decays were fitted with a mono-exponential function to obtain the minority carrier lifetimes. Both PL and time-resolved PL decays were measured along the length (x, μm) of the nanowires. The tip where Au particle sits is defined as the top of the nanowire (*x* = 0 μm). The bottom of the nanowire was determined by where it broke from the substrate. Between 8 and 10 nanowires were measured for each sample and results were consistent across all wires. Positions of the measured nanowires were marked and studied by scanning electron microscopy (SEM) to determine the position of PL emission relative to the tip of the nanowires.

## Results and Discussion

**Figure 1:**
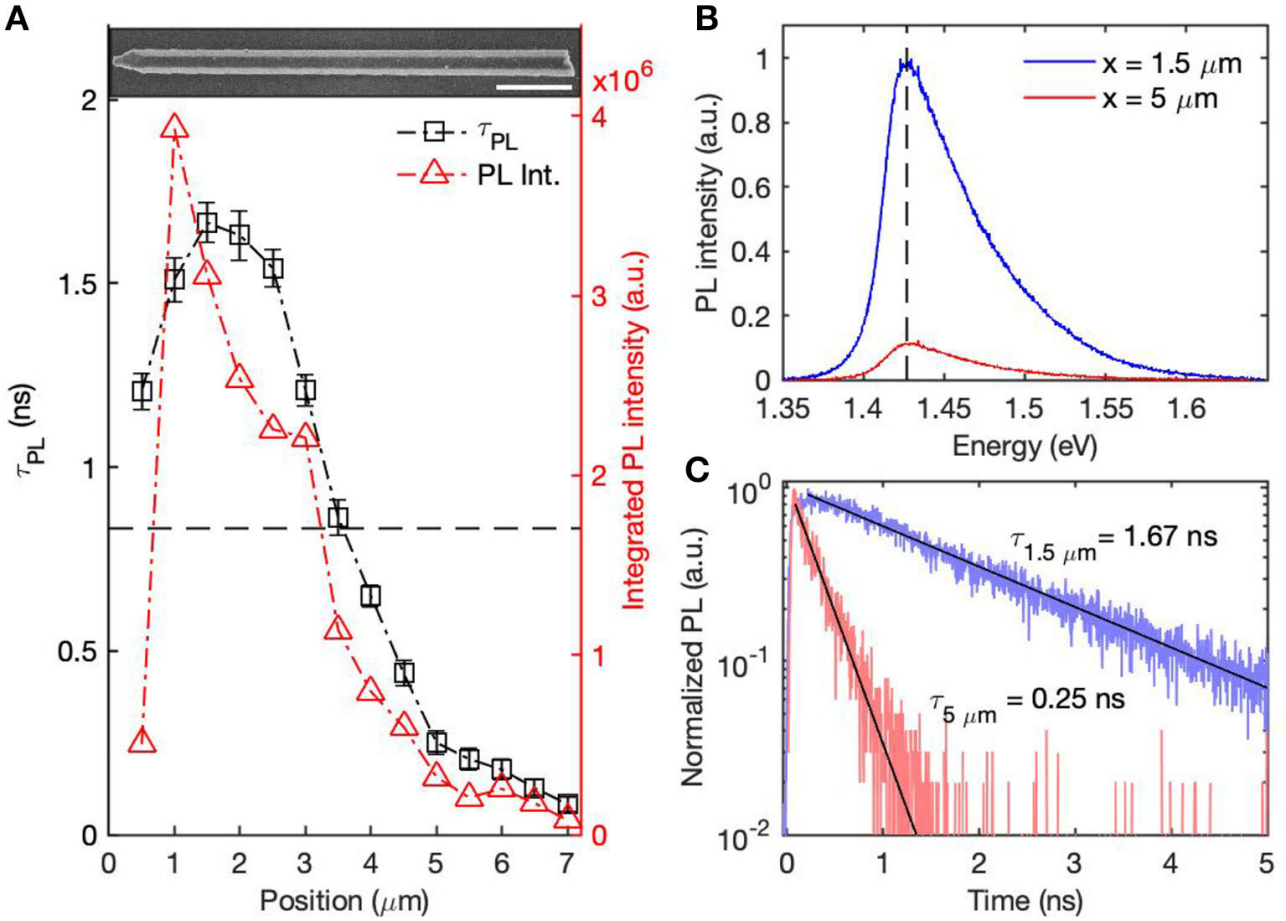
Room temperature PL measurements from a single GaAs/AlGaAs core-shell nanowire with 100 nm GaAs core. **(A)** PL intensities and lifetimes measured along the nanowire, as shown in red triangles and black squares respectively. The inset shows an SEM image of the nanowire. The scale bar is 1 μm. **(B)** PL spectra selected from the top and bottom of the nanowire with band edge emission 1.43 eV marked. **(C)** The time-resolved PL decays measured at 1.43 eV corresponding to the PL spectra in **(B)**. Mono-exponential fits are shown with lifetimes displayed.

Figure 1A shows the PL measurements from a single GaAs/AlGaAs core–shell nanowire (100 nm GaAs core) with an SEM image of the same nanowire inset. The nanowire shows minimal tapering along its length, except in the region next to the Au nanoparticle sitting on the top. The slanted facets adjacent to the Au nanoparticle are {111}, and are formed during the AlGaAs shell and GaAs cap growth, rather than during GaAs core growth. Their impact on the PL emission from the GaAs core is therefore negligible. Despite the apparently uniform morphology, the nanowire shows significantly greater emission (higher PL intensity and longer lifetime) from the upper half nearer the Au nanoparticle, and less emission (lower PL intensity and shorter lifetime) from the bottom half. A PL lifetime of 1.21 ns was measured at the top of the nanowire, increasing to a maximum of 1.67 ns at a distance of *x* = 1 μm, followed by a subsequent drop. The top (2.5 ±0.5) μ*m*, approximately half of the wire, shows lifetimes above 1 ns. Lifetimes <100 ps were measured toward the bottom end of the nanowire. The integrated PL intensities follow a similar trend to the PL lifetimes with brighter PL emission corresponding to longer lifetimes.

Typical PL spectra and time-resolved PL decays were selected from the top and the bottom half of the nanowires, which, respectively, represent regions of higher and lower optoelectronic quality, as shown in Figures 1B,C. Despite the dramatic change in PL intensity, band edge emission at 1.43 eV was observed along the whole nanowire with no obvious red-shift. All time-resolved PL decays were mono-exponential, indicating that the recombination processes were dominated by Shockley-Read-Hall recombination in all locations.

**Figure 2:**
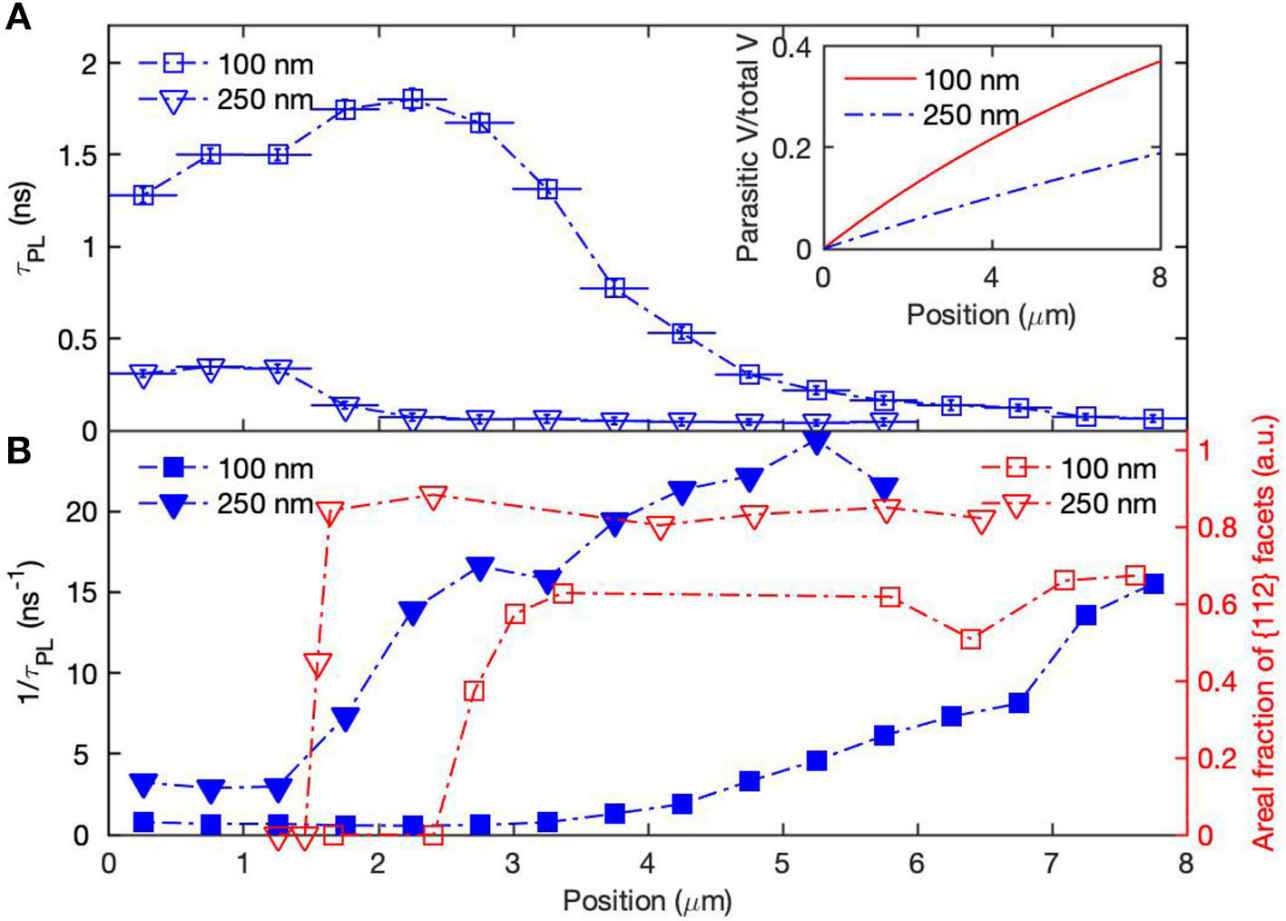
**(A)** A comparison between the τ_*PL*_ measured along two GaAs/AlGaAs core-shell nanowires with 100 and 250 nm GaAs cores. The inset shows the volume fraction of parasitic growth over total GaAs volume along the nanowires with different diameters. **(B)** The recombination rate, $\frac{1}{\tau_{PL}}$, (blue) and the fraction of surface area (areal fraction) occupied by {112} facets as a function of position, x, calculated from the SEM images.

To investigate the causes for the non-uniform PL behavior, we compared the lifetimes for the core–shell nanowires with 100 and 250 nm GaAs cores, as shown in Figure 2A. The 250 nm cores show a trend similar to that of the 100 nm cores; both exhibit a high-quality upper half and a low-quality lower half. However, lifetimes measured from the 250 nm cores are much shorter than those measured from the 100 nm cores. Furthermore, the length of the high-quality segment of the 250 nm cores is much shorter than that of the 100 nm cores.

Considering the possible carrier recombination paths, lifetime τ_*PL*_ can be expressed as

$$\begin{array}{l} \frac{1}{\tau_{PL}}=\;\frac{1}{\tau_{r}}+\;\frac{1}{\tau_{non,b}}+\;\frac{4S}{d} \end{array}$$

where τ_*r*_ is the radiative recombination lifetime, τ_*non, b*_ is the non-radiative recombination lifetime in the bulk, *S* is the surface recombination velocity at the GaAs/AlGaAs core–shell interface, and *d* is the diameter of the nanowire's GaAs core. The radiative recombination lifetime τ_*r*_ can be calculated as 1/BN, where B as the radiative recombination coefficient and N is the carrier density. Assuming a unity internal quantum efficiency in carrier generation (i.e., one electron-hole pair is generated for each absorbed photon), B = 7.2 × 10^−10^ cm^3^/s (Varshni, 1967), and absorption coefficients of 5.3 × 10^4^ cm^−1^ for GaAs and 9.0 × 10^4^ cm^−1^ for AlGaAs at 522 nm (Aspnes et al., 1986), a lower limit of τ_*r*_ is estimated to be 25–250 ns for the 100 nm cores [with excitation fluence of 0.2–2 μJ/(pulse^*^cm^2^)]and 10 ns for the 250 nm cores [with excitation fluence of 14 μJ/(pulse^*^cm^2^)]. The higher fluences were used to generate enough signal at the bottom of the nanowires, particularly for the nanowires with 250 nm cores. The significantly longer calculated τ_*r*_, compared with the measured PL lifetimes, suggests the process is dominated by the non-radiative recombination.

One possible explanation for the poor PL lifetime toward the bases of the nanowires is that the parasitic radial GaAs growth at the nanowire base incorporates a significant proportion of non-radiative recombination centers (Bolinsson et al., 2014). This parasitic radial growth occurs as the nanowire elongates, and causes the cores to exhibit a tapered morphology because the bases are exposed to radial growth longer than the more recently grown tips. To estimate the contribution of this undesirable radial GaAs material, we calculate the degree of tapering and the fractional volume of the GaAs core occupied by this material.

Tapering of the as-grown GaAs nanowires was measured by SEM, and is quantified as the increase in diameter per unit of nanowire length. The tapering of the 100 and 250 nm-diameter GaAs nanowires was very similar, at 3 ± 1 *nm*/μ*m*. The proportional volumes of this parasitic growth relative to the total GaAs volume for both nanowires are shown in the inset of Figure 2A. If parasitic radial growth is the cause of the degradation in PL lifetimes, the effect should be much more significant for the 100 nm-diameter wires. For example, at a position 4 μm from the nanowire tip, the parasitic radial material contributes 21% to the overall GaAs volume in a nominally 100 nm-diameter core, in contrast to 10% for a nominally 250 nm-diameter core. To investigate this possibility, we calculate the ratio τ_x = 4μ*m*_/τ_x < 1μ*m*_ for the two different nanowires, yielding a value of ~0.3 for both samples. As there is no significant difference in the ratio between the two diameters, we conclude that parasitic radial growth cannot completely explain the variation in PL lifetime along the nanowire length. Furthermore, the parasitic radial growth depends linearly on the distance from the nanowire tip. It does not show the same abrupt changes with length as seen in the plot of τ_*PL*_ against length. This lack of correlation between tapering and τ_*PL*_ indicates that parasitic radial growth is not likely to be the main cause for the change of τ_*PL*_ along the length of the nanowire.

We next considered the impact of the free surface created at the base of nanowires when broken from the host substrate and transferred onto Si substrate. As this surface is unpassivated, it may act as a non-radiative recombination sink for carriers in its vicinity which may contribute to the poor PL emission and short τ_*PL*_ toward the bottom end of the nanowire. If this effect was dominant, we would expect the charge carrier density along the nanowire and hence the PL along the nanowire, to follow a one-dimensional diffusion model that is independent of the core diameter. That is, we would expect that the 100 and 250 nm-diameter wires would show near-identical dependences of τ_*PL*_ vs. length, with only minor variation between diameters due to the higher carrier mobility of the larger diameter nanowires (Joyce et al., 2017). However, in Figure 2A, we observe marked differences between the two diameters, which suggests that recombination at the bottom free surface cannot be solely responsible for the dependence of τ_*PL*_ on length. To examine this effect further, we performed additional experiments using a pinhole in the PL collection optics for confocal detection. The pinhole restricted the PL signals collected to a ~1μ*m* segment along the nanowire, centered at the excitation spot. PL data for the 250 nm-diameter core–shell nanowires, measured with and without the pinhole, are presented in Supplementary Figure 2. No significant differences were observed between the two set of τ_*PL*_, which indicates that the carrier diffusion length is <1 μ*m*. With such a short carrier diffusion length, it is unlikely that recombination at the unpassivated base significantly affects PL from regions lying more than 1 μ*m* distance from the nanowire base.

Another parameter that changes along the length of the GaAs nanowires is the orientation of the sidewall facets. In our earlier report, we showed that the GaAs nanowires go through complicated sidewall facet changes during the two-temperature core growth and ramping up to higher temperatures for subsequent AlGaAs shell growth (Jiang et al., 2014). As a result, the nanowires exhibit different facets along the length of the nanowire—{110} facets at the top and a mixture of {110} and {112} facets toward the bottom. Planar AlGaAs passivated GaAs {110} surfaces in the form of GaAs quantum wells are widely reported, however, such reports on the same structures with of GaAs {112} are rare. This may be related to the unstable nature of {112} planes at high temperatures. The nanowire geometry, presenting both {110} and {112} facets, allows us to ascertain the effect of these facets.

Theoretical calculations show that intrinsic surface state density of GaAs is determined by the surface atom structure (Ivanov et al., 1980) and is highly dependent on the orientation. For example, the {110} surface is free of intrinsic mid-gap surface states (Spicer et al., 1977) whereas the mid-gap surface state density of both {100} surface and {111}B were reported to be high (e.g., for {100} it can reach 5 × 10^11^ cm^−2^) (Offsey et al., 1986; Miller and Richmond, 1997) {112} can be considered to be comprised of {111} and {001} at a ratio of 2:1 (Joyce et al., 2010). For a free surface, this drastic difference is typically overwhelmed by the surface states introduced by oxygen (Spicer et al., 1977). Indeed, studies on bare GaAs nanowires showed near-identical surface recombination at uncapped {110} and {112} surfaces (Joyce et al., 2014). Nonetheless, the difference of the intrinsic surface state density may be revealed at a well-passivated surface. It has also been widely reported in GaAs/AlGaAs planar structures that the orientation of the growth surfaces has a significant impact on the AlGaAs quality and hence the GaAs/AlGaAs interface quality (Harrison et al., 1978; Fukunaga et al., 1987; Nilsson et al., 1989; Chand, 1993). One of the major concerns is oxygen incorporation which is associated with deep non-radiative traps (Kuech et al., 1987). Oxygen readily incorporates to AlGaAs (Islam et al., 1995), and one oxygen atom in thousands of surface atoms is sufficient to create enough surface states to pin the Fermi level and increase the recombination velocity significantly. Yet, oxygen tends to incorporate on As-rich surfaces where the sticking coefficient for oxygen is higher (Spicer et al., 1977). Indeed, early studies show that oxygen incorporation on {112} facets, in particular {112}B, was greater than that on {110} (Ranke et al., 1982; Chand, 1993). Due to the significant impact of oxygen, a small difference in oxygen incorporation on {110} and {112} surfaces may lead to the observed significant difference in surface recombination velocity.

**Figure 3:**
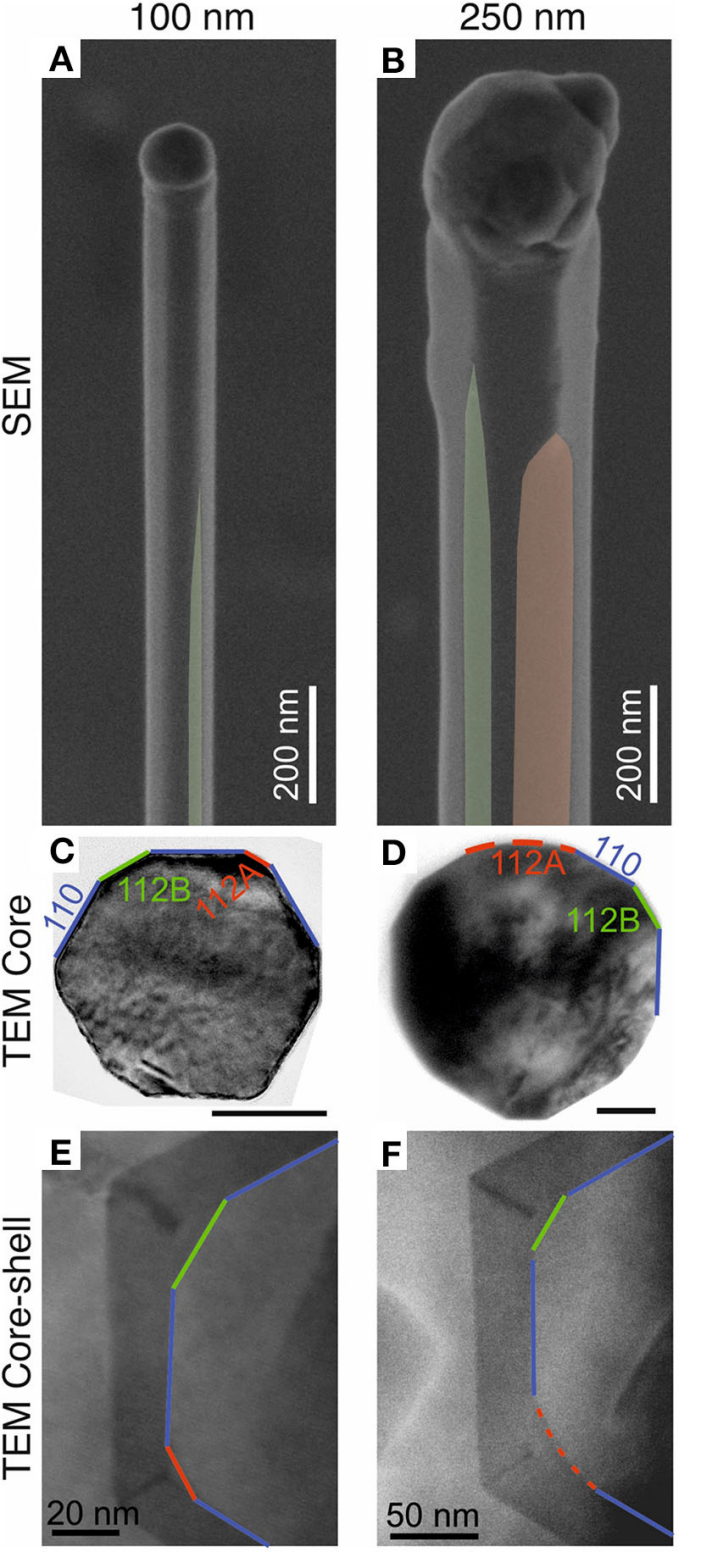
Geometries of GaAs nanowires with the diameters of **(A)** 100 nm and **(B)** 250 nm after annealing at 750°C for 2 min with {112}A planes highlighted in red and {112}B planes highlighted in green. **(C,D)** show bright field TEM images of cross-section taken from the bottom half of the nanowires with diameters of **(C)** 100 nm and **(D)** 250 nm. The scale bars in **(C,D)** are 50 nm. The high index curved surface toward {112}A is highlighted by the red dashed line in **(D)**. The bend-contours observed in **(C,D)** were artifacts caused by the cross-sectional TEM sample preparation, but do not affect the nature of the as-grown facets. **(E,F)** compare the GaAs/AlGaAs interface for nanowires with two different core sizes of **(E)** 100 nm and **(F)** 250 nm. For the TEM images, {112}A, {112}B, and {110} planes are highlighted in red, green and purple lines, respectively. The curved planes toward {112}A is highlighted by red dashed lines in **(D,F)**.

To investigate the impact of sidewall facet change, the facets of the GaAs core nanowires were studied by both SEM and cross-sectional TEM before and after AlGaAs shell growth, as shown in Figure 3. It is clearly seen in Figures 3A,B that both the 100 and 250 nm GaAs nanowire cores show {110} facets at the top of the nanowires which change to a mixture of {110} and {112} facets toward the bottom of the nanowires. Measurements using SEM show the lengths of the segment with purely {110} facets are (720 ± 70) nm and (250 ± 20) nm for 100 and 250 nm nanowires, respectively. The difference in standard deviation for the two set of nanowires is because it is more difficult to resolve {112} facets for the 100 nm nanowires in SEM. The facets toward the bottom of the nanowires were identified by cross-sectional TEM (Figures 3C,D) using the same method as reported in our earlier work (Jiang et al., 2014). While the 100 nm core shows a mixture of {110}, {112}A, and {112}B facets, the high-index curved surfaces (Figure 3D) remained for 250 nm cores even after being subjected to temperatures of 750°C. Those facets were well-preserved during the AlGaAs shell growth, as shown in Figures 3E,F. We noted that the {112}A facets for the 250 nm cores were bigger than those of the 100 nm cores. This is largely caused by the geometry of the nanowires, as explained in detail in Supplementary Material. We measured the {112} facet ratios (width of {112} facets/perimeter of the nanowire) along the length of the nanowires after annealing at 750°C and present them Figure 2B. Figure 2B shows that the {112} facet ratios for both the 100 and 250 nm cores mirror the trend in recombination rate $\left(\frac{1}{\tau_{PL}}\right)$. The ratio of {112} facets for the 250 nm cores is significantly higher than that for the 100 nm nanowires, which is likely the cause of the much shorter τ_*PL*_ measured from the core–shell nanowires with 250 nm GaAs cores. For the 100 nm cores, we observe a continuing decrease in τ_PL_ after the {112} facet ratios have stabilized toward the nanowire bases. It is likely due to a combination of factors discussed above, including surface evaporation of As species that is more severe toward the bottom of the nanowires (Jiang et al., 2014), non-radiative recombination sites in the parasitic radial growth that occurs during low-temperature core growth, and non-radiative recombination at the uncapped nanowire base. Nevertheless, these factors are secondary to the effect of the {112} facets.

**Figure 4:**
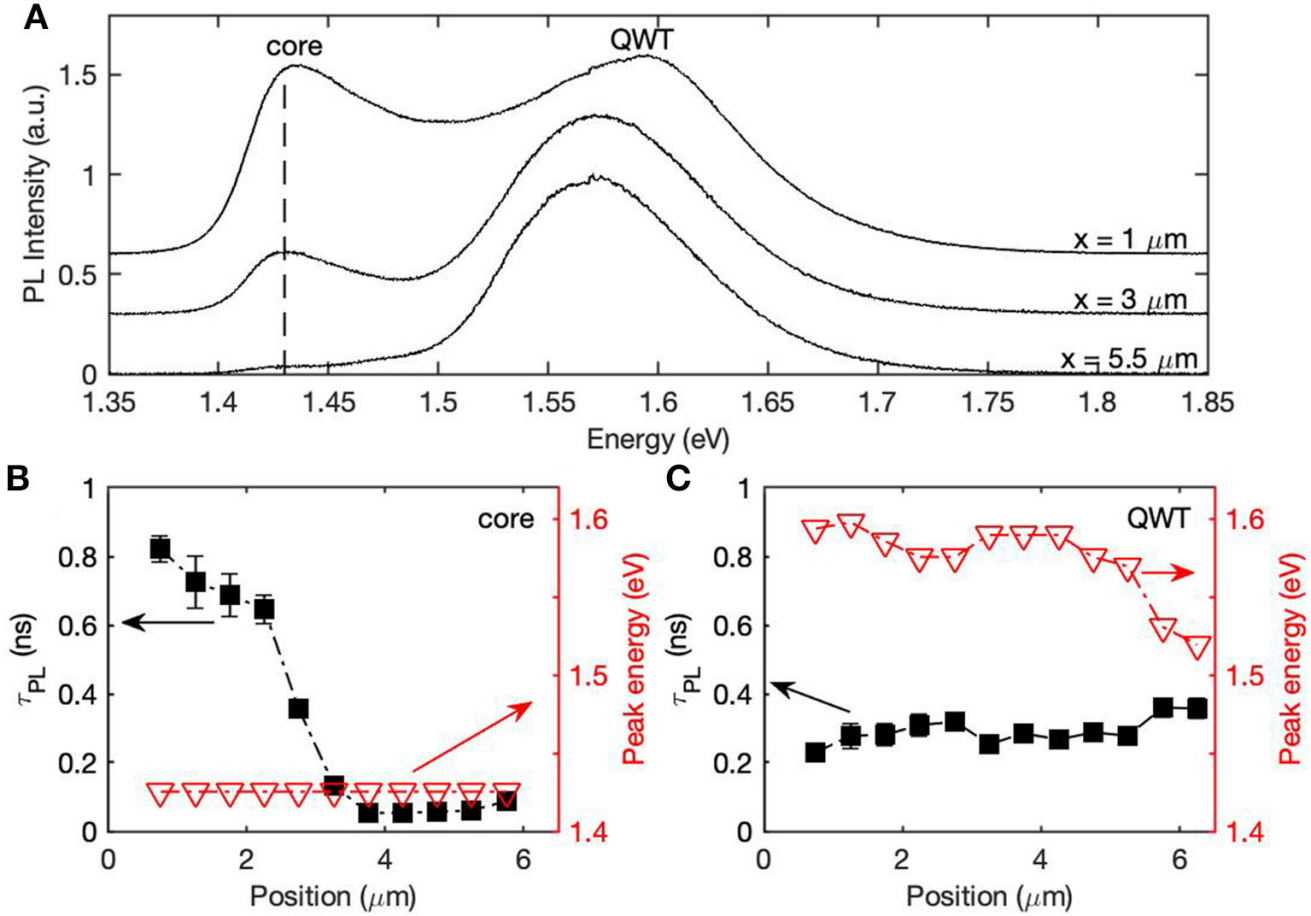
**(A)** PL spectra selected from the top, middle and bottom of a quantum well tube (QWT) nanowire. **(B,C)** compare the τ_PL_ and PL peak energies of the **(B)** GaAs core and **(C)** the GaAs QWT.

To prevent both the facet change and surface evaporation, we prepared the GaAs/AlGaAs quantum well tube nanowires (Supplementary Figure 4) using a recipe previously reported (Shi et al., 2015). The thickness of quantum well tube was chosen so that the PL emissions from GaAs core and the GaAs quantum well tube were spectrally separated at room temperature. The GaAs tube is grown upon an AlGaAs shell which naturally presents {110} facets, and the high growth temperature of the GaAs tube promotes the formation of {110} facets at its surface. The facets are therefore {110} for the entire length of the GaAs quantum well tube. Figure 4 shows the PL spectra and carrier lifetimes measured at the peak emission energies from both the GaAs core and quantum well tube. It is clear that the emission of the GaAs quantum well tube is more uniform than that of the GaAs core. Carriers within the quantum well tubes experience strong confinement, which increases the radiative recombination rate from these tubes and accounts for the charge carrier lifetime of ~0.3 ns. The slight change of τ_PL_ from the quantum tube emission along the nanowire is correlated to the change of peak energy which is mostly caused by the quantum well thickness variation.

We thus conclude that the non-uniform PL emission of the GaAs/AlGaAs core–shell nanowires and the unusual diameter dependence of τ_*PL*_ are mainly caused by the orientation change of the sidewall facets of the GaAs core nanowires. We observe that {110} facets give rise to superior PL lifetime and intensity, whereas {112} facets that spontaneously form toward the nanowire bases are associated with poorer optoelectronic properties. We propose a radial quantum well tube heterostructure to avoid this non-uniformity in optical properties. Although observing nanowire sidewall facets along the length can be challenging, this study shows the significant impact of sidewall facets on the nanowire optoelectronic properties. Thus, knowing the nanowire sidewall facets is important to simulate and understand the performance of nanowire devices, in particular for the nanowires grown by vapor-liquid-solid mechanism where complicated sidewall changes might be involved.

## Data Availability Statement

The raw data supporting the conclusions of this article will be made available by the authors, without undue reservation.

## Author Contributions

NJ designed and carried out the nanowire growth and characterization experiments, prepared the manuscript draft, and revised the manuscript. HJ edited and revised the manuscript. NJ, HJ, and PP participated in data analysis and results discussion related to the spectroscopy. NJ, HJ, and JW-L participated in results discussion related to nanowire structures. NJ, HJ, HT, and CJ participated in results discussion related to nanowire growth. All authors contributed to the article and approved the submitted version.

## Conflict of Interest

The authors declare that the research was conducted in the absence of any commercial or financial relationships that could be construed as a potential conflict of interest. The handling editor declared a past co-authorship with the authors PP, HT, and CJ.
