# Supplementary material for: Facet-Related Non-uniform Photoluminescence in Passivated GaAs Nanowires
Source: Front Chem. 2020 Dec 7;8:607481. doi: 10.3389/fchem.2020.607481 (PMC7750184; doi:10.3389/fchem.2020.607481)
Supplement: Supplementary file 1 [file Table_1.DOCX]

Supplementary Information for

Facet-related Non-uniform Photoluminescence in GaAs nanowires


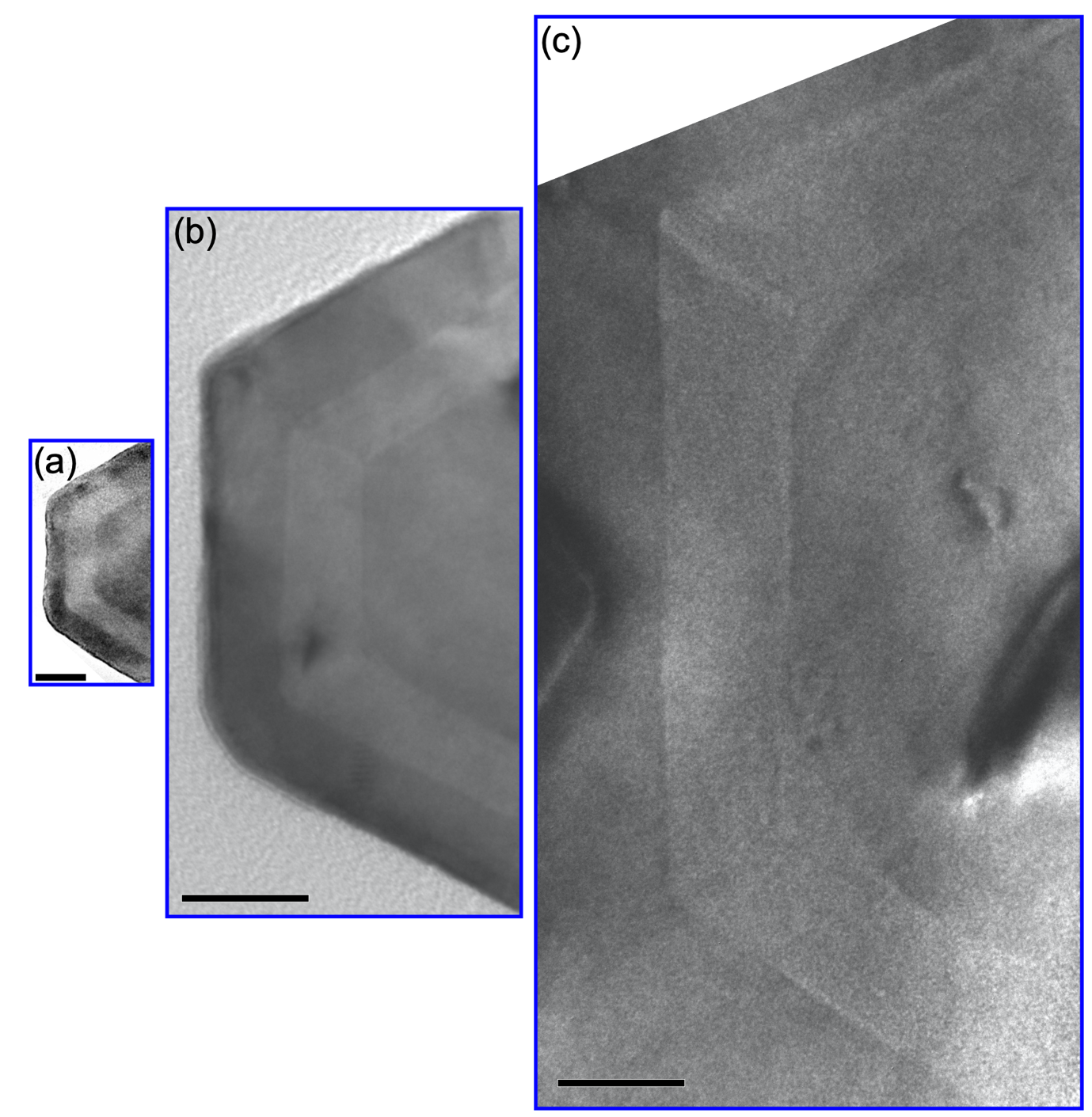


Figure S1. Cross-sectional TEM micrographs of GaAs/AlGaAs core-shell nanowires with 3 min AlGaAs shell growth and with core diameters of (a) 50 nm, (b) 100 nm and (c) 250 nm. Scale bars are 20 nm in (a) and 50 nm in (b-c).


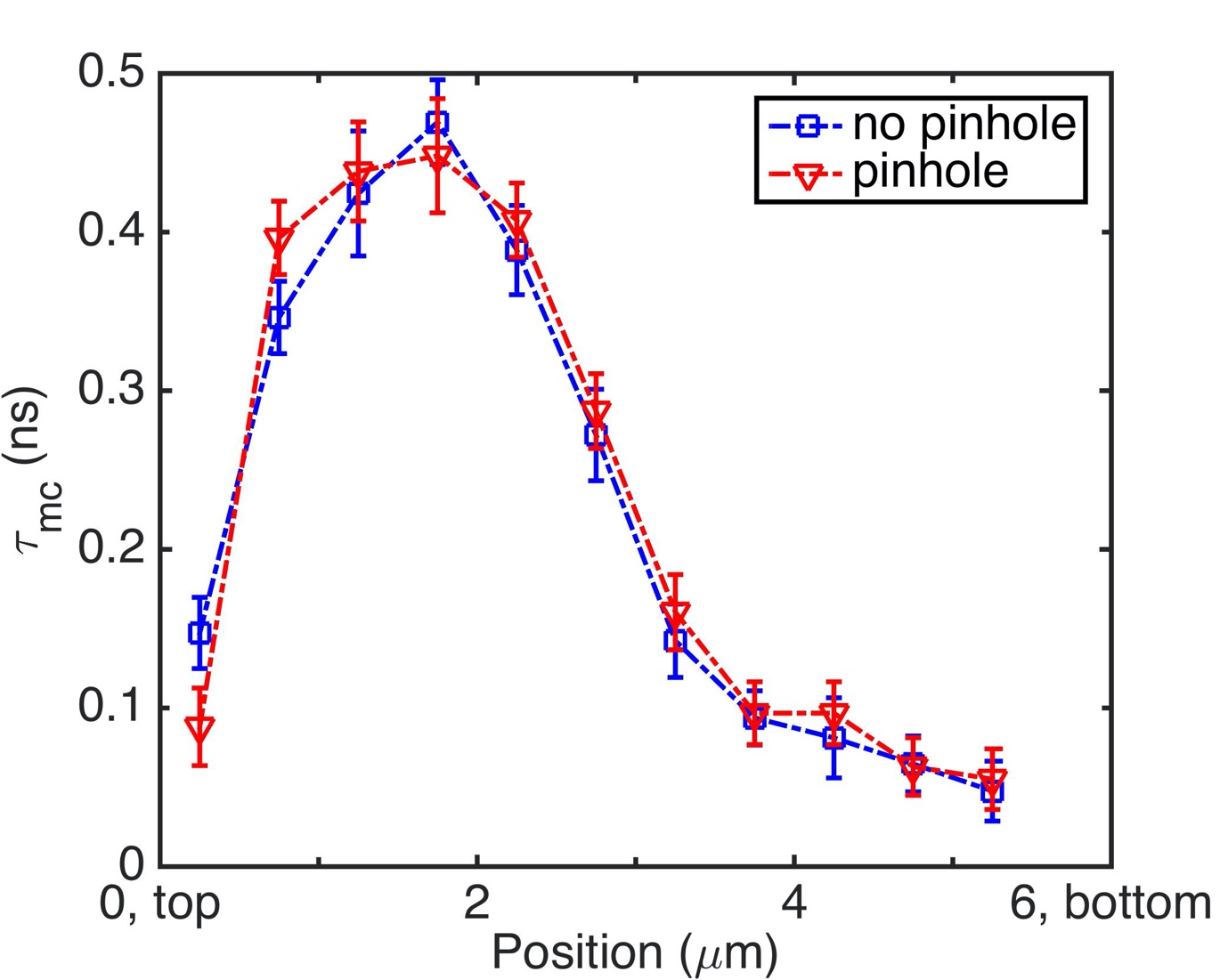


Figure S2. Comparison of $\tau_{PL}$ measured along a GaAs/GaAs core-shell nanowire with and without a pinhole in front of the monochromator. The diameter of the GaAs core was 250 nm. The pinhole (red triangles) limited the collected PL signal to that emitted from a length of 1 $\mu m$ centred at the excitation spot whereas without the pinhole (blue squares), the PL signal was collected from the whole nanowire.


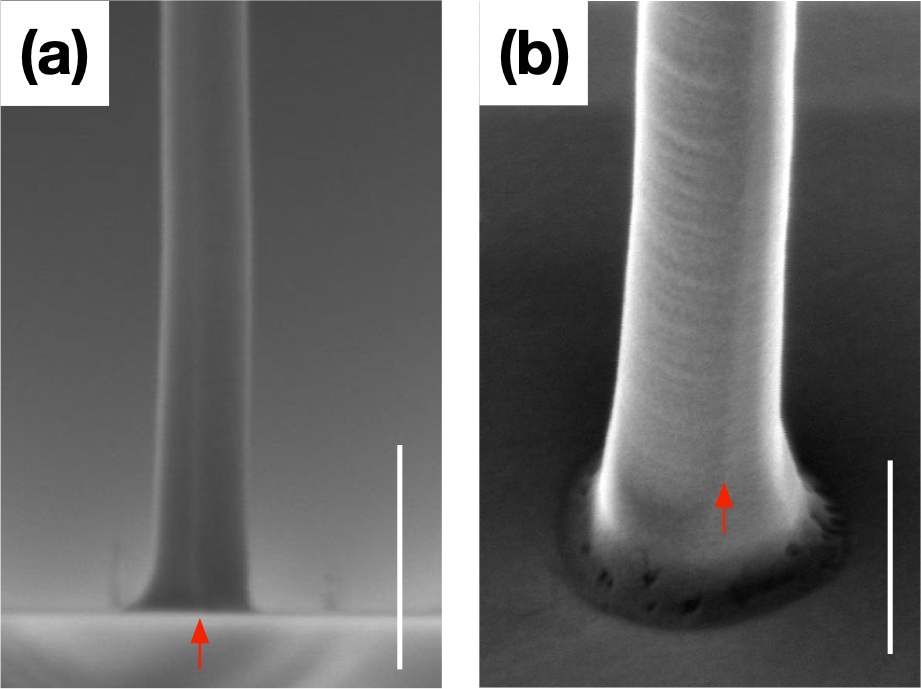


Figure S3. SEM images of as-grown GaAs nanowires with diameters of (a) 100 nm and (b) 250 nm. The arrows point to the intersection where {112}A (or curved surface towards {112}A) meet {112}B. Scale bars are 300 nm. For the 100 nm nanowire, the electron beam is aligned to <110> direction. The intersection is near the centre of the nanowire, indicating the cross-sectional shape is close to a hexagon. For the 250 nm nanowire, the sample was tilted slightly so that the {112}B sidewall facet can be resolved clearly. The intersection is close to one side of the nanowire, indicating the cross-sectional shape is a truncated Reuleaux triangle with elongated facets curved towards {112}A and shorter {112}B facets.

**SI. Facet changing of GaAs nanowires with diameters of 100 nm and 250 nm**

GaAs nanowires with 250 nm diameters present more extended {112}A facets, compared to those with 100 nm cores which show a greater dominance of {110} facets. The reasons for this are related to the processes taking place during (a) core growth and (b) annealing, as follows.

1. During the nanowire growth:

Our previous work^1^ has shown than in the immediate vicinity of the Au nanoparticle, the nanowire cross-section initially forms in the shape of a Reuleaux triangle, with elongated curved high-index facets that approach {112}A facets, and shorter curved facets that approach {112}B facets. Parasitic radial growth then causes the cross-section to evolve due towards a more hexagonal shape made of both {112}A and {112}B facets. The amount of radial growth required to elicit this change is related to the nanowire diameter: 250 nm-diameter wires require a larger volume of radially deposited material, whereas 100 nm-diameter wires require a smaller amount of radial growth to adopt a perfect hexagonal shape. Our growth conditions are chosen to minimise parasitic radial growth. This causes the differences of the sidewall facets of the as-grown nanowires: the sidewall facets for 250 nm as-grown nanowires are mostly high index surfaces towards {112}A with small {112}B facets, whereas the sidewall facets for 100 nm as-grown nanowires are closer to perfect hexagonal shape made of both {112}A and {112}B facets. (Figure S3)

1. During the temperature ramp-up:

As the temperature is ramped-up (prior to shell growth), surface atoms migrate to adopt a more energetically stable shape with dominant {110} facets. (Ref 23) This transition is affected by the diameter of the nanowire, and the facets initially presented by the nanowire:

- The average migration distance per atom, required to change to {110} facets for a Reuleaux triangle is almost twice as much as that for a hexagon made of {112} facets, and is also proportional to the radius of the nanowires.^1^
- The amount of volume required to change to {110} facets for a Reuleaux triangle or truncated Reuleaux triangle is 2-3 times more than that for a hexagon, and is proportional to the square of radius of the nanowires.

As a result, it is much more difficult for the 250 nm nanowires to change their shape and their original shape is better preserved through the nanowire growth process. Hence, the {112}A facets appeared larger in the 250 m cores than that in the 100 nm cores.


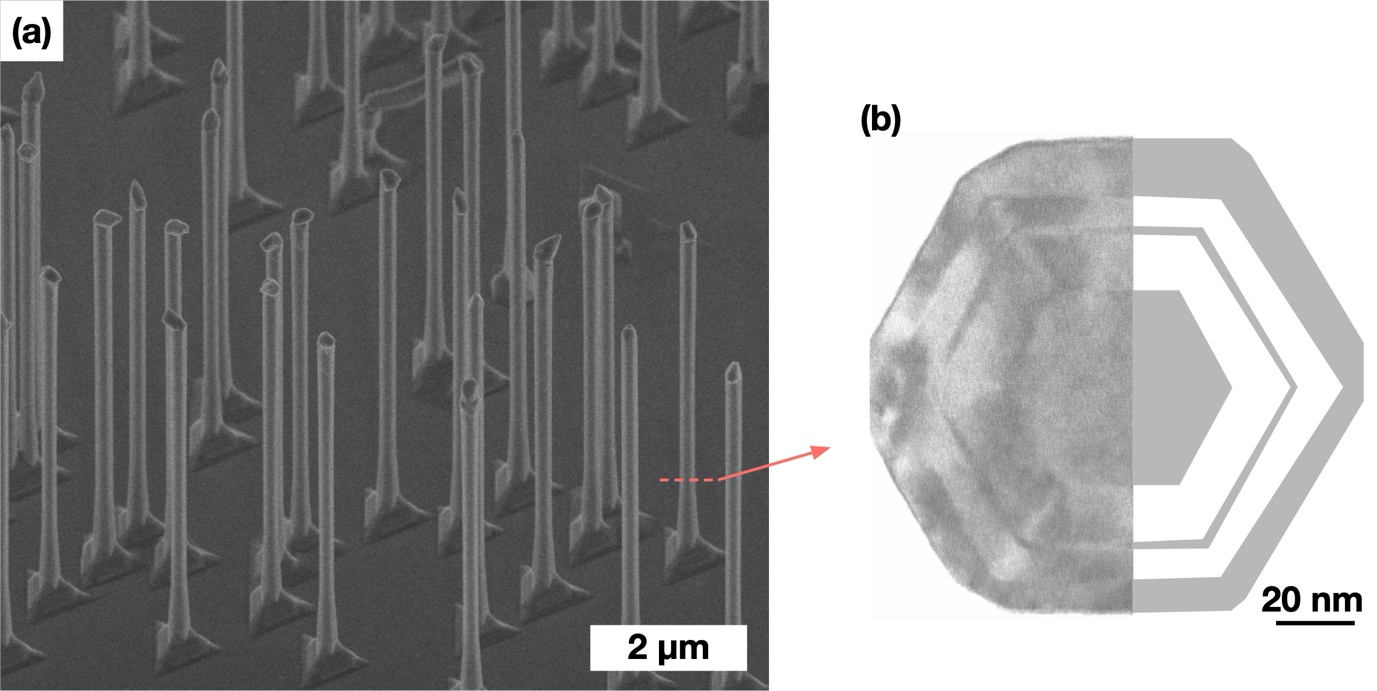


Figure S4. (a) SEM image of the GaAs/AlGaAs quantum well tube nanowires. (b) bright field cross-sectional TEM image with the layer structure illustrated.

**Reference**

1. Jiang, N. *et al.* Understanding the true shape of Au-catalyzed GaAs nanowires. *Nano Lett.* **14**, 5865–5872 (2014).
